# 'Foraging' for a place to lay eggs: A genetic link between foraging behaviour and oviposition preferences

**DOI:** 10.1371/journal.pone.0179362

**Published:** 2017-06-16

**Authors:** Murray W. McConnell, Mark J. Fitzpatrick

**Affiliations:** 1 Integrative Behaviour & Neuroscience Group, Department of Biological Sciences, University of Toronto Scarborough, Toronto, ON, Canada; 2 Department of Ecology & Evolutionary Biology, University of Toronto, Toronto, ON, Canada; 3 Department of Cell & Systems Biology, University of Toronto, Toronto, ON, Canada; Biomedical Sciences Research Center Alexander Fleming, GREECE

## Abstract

Gravid female arthropods in search of egg-laying substrates embark on foraging-like forays: they survey the environment assessing multiple patches, tasting each with their tarsi and proboscis, and then, if interested, they deposit an egg (or eggs). In fruit flies, *Drosophila melanogaster*, allelic variation in the *foraging* gene (*for*) underlies the rover/sitter foraging behaviour polymorphism. Rover flies (*for*^R^) are more active foragers (both within and between food patches) compared to sitters (*for*^s^). In nematodes, *Caenorhabditis elegans*, a mutation in *egl-4*, the ortholog of *for*, leads to aberrations in egg laying. Given this and the notion that females may ‘forage’ for a place to oviposit, we hypothesized that *for* may underlie egg-laying decisions in the fruit fly. Indeed, when given a choice between patches of low- and high-nutrient availability, rovers lay significantly more eggs on the low-nutrient patches than sitters and also a sitter mutant (*for*^s2^). We confirm the role of *for* by inducing rover-like oviposition preferences in a sitter fly using the transgenic overexpression of *for*-mRNA in the nervous system.

## Introduction

The selection of a suitable location for oviposition by females can have measurable implications on the nutrition and survival of their offspring [1]. In many species, especially arthropods, this represents one of the only opportunities for ‘parental care.’ Evidence suggests that preferred oviposition substrates include those of high resource quality and enemy-free spaces [2–4]. In *Drosophila* spp. oviposition site selection (OSS) is influenced by many factors including alcohol content [5,6], inherited preferences [7], geotactic tendencies [8], previously occupied food sources [9–11], food texture [12], availability [13] and proximity [14] of substrates, social cues [15] and perceived larval foraging costs [16]. Recently, Yang *et al*. [17] show *D*. *melanogaster* flies prefer to oviposit on substrates with lower sugar concentrations. Yang *et al*. [17] also provide evidence that *Drosophila* females probe each site with her proboscis and lay eggs on an egg-by-egg basis (i.e. not in batches), suggesting that females are constantly assessing their surroundings for suitable oviposition substrates and appear to do so using foraging-like behaviours. Although foraging and oviposition behaviours have been linked previously [18,19], the genetic underpinnings of natural variation in oviposition strategies is unknown.

First characterized by Sokolowski [20], naturally occurring allelic variation in *for* was initially shown to affect larval foraging behaviour. Larvae with the *for*^*R*^ allele (called rovers) travel greater distances within and between food patches than individuals homozygous for the *for*^*s*^ allele (called sitters) [20–22]. Using reciprocal crosses, *for* was localized to the 2^nd^ chromosomes [23] and it was later found to encode a cGMP-dependent protein kinase (PKG). Rovers have greater mRNA transcript levels and PKG activity than sitters [24]. Aside from larval foraging, natural variation in *for* also influences other phenotypes including adult foraging behaviour [25, 26], dispersal [27], pupation site selection [28, 29], sucrose responsiveness and habituation [30, 31], food intake/energy homeostasis [32], learning and memory in larvae [33] and adults [34], and stress tolerance [35, 36].

The role of *for* in food-related behaviours may be evolutionarily conserved [37] with formal associations in bees (honeybee: [38], bumblebee [39]), nematode worms (*C*. *elegans*: [40]; *Pristioncus pacificus*: [41]), and ants (*Pogonomyrmex barbatus*: [42]; *Phediole pallidula*: [43]). Interestingly, in *C*. *elegans*, *egl-4*, the ortholog of *for*, was originally described to affect egg-laying [44]. Therefore, given this and the fact that OSS contains many elements similar to foraging behaviour, we hypothesized that natural allelic variation in *for* influences OSS in *D*. *melanogaster*. Based on classical approaches to studying foraging behaviour, we varied habitat quality to elicit potential differential responses in the egg-laying preferences of rovers and sitters.

## Materials and methods

### (a) Fly stocks

Rover and sitter strains are homozygous for the *for*^*R*^ and *for*^*s*^ alleles, respectively, on the second chromosomes and share co-isogenic third chromosomes from the *for*^*R*^ strain [20]. *for*^*s2*^ is a sitter-mutant strain that was generated on a rover genetic background with an induced mutation at the *foraging* locus which confers sitter-like *for* mRNA-expression, PKG activity levels, and foraging behaviour [24, 25, 45]. The three strains used in the transgenic overexpression crosses (*w*^1^; *for*^s^; *elav*-GAL4, *w*^1^; *for*^s^; UAS-*for*T1a, and *w*^1^; *for*^s^; +) were obtained from M. Sokolowski. The GFP flies used in the preseeding experiment (+; *for*^s^; *Ubi*-GFP and +; *for*^R^; *Ubi-*GFP) were previously described in [46].

### (b) Fly rearing conditions and media

Fly stocks were maintained in 170 mL plastic culture bottles (VWR) with 40 mL of standard culture medium and were reared at 23 ± 1°C, and 65 ± 5% relative humidity, and a 12L:12D photocycle (lights off at 1900 h). One litre of standard culture medium contained 50 g of active dry yeast (Fleischmann’s), 100 g of sucrose, 16 g of agar, 0.1 g of KH_2_PO_4_, 8 g of C_4_H_4_KNaO_6_, 0.5 g of NaCl, 0.5 g MgCl_2_, and 0.5g Fe_2_(SO_4_)_3_.

### (c) Nutrient-adjusted food patches for oviposition

To approximate naturally occurring heterogeneity in substrate quality, we exposed females to ‘patches’ that varied in nutrient abundance (low and high). The high-nutrient food represents the standard culture media described above whereas the low-nutrient food contains an 85% reduction in the yeast (protein source) and sugar (carbohydrate source) components of the standard media [32, 46]. Larvae reared on this low-nutrient diet have been shown to be significantly smaller at third instar and have increased development time than those reared on standard fly media [32]. Experimental media was darkened using powdered charcoal (0.0026 mg/L), a non-nutritive addition that facilitated egg counting [47].

### (d) Isolating flies for oviposition experiments

Founding flies were removed from the stock bottles prior to the eclosion of their offspring. Each day thereafter, newly emerged adult flies were removed from the stock bottles and placed in new vials (50 mL, VWR, containing 10 mL of media) overnight. The following day, these flies were subdivided into vials that ultimately housed 64 females and 20 males until one day prior to the experiment. The establishment of these vials served two purposes: i) it facilitated the preparation for the oviposition assay (64 females), and ii) fewer flies and a 3:1 ratio of females to males allows for mating without excessive stressing of females by males (MJF, pers obs). Males were removed from the vials after 24 hours. All females were 3 day old adults when assayed.

### (e) Experimental arena & setup

Oviposition arenas consisted of translucent plastic containers (11 cm × 11 cm × 5.5 cm, Dollarama). A sponge was placed in a hole cut in the centre of the lid to allow for sufficient air exchange and the transferring of flies into the container. The food patches for oviposition consisted of the caps from 1.5 mL microcentrifuge tubes (Axygen, VWR) filled with media. Each trial is a binary comparison of food types with two microcentrifuge caps filled with each type. Two ‘patches’ per food type allowed us to account for any possible aggregation tendencies. The filled caps were placed randomly (coin flip) at the corners of an unmarked 4 cm × 4 cm square centered on the bottom of the testing arena and were secured using small blobs of plastiscine. For each individual trial, one food type was marked with a small dot from a black felt pen (Sharpie) and this was determined randomly by a coin flip. Between 15:30 and 16:00 h the females were placed into the testing arenas which are then immediately placed in the incubator 25 ± 1°C and 65 ± 5% relative humidity. Flies were housed in the incubator overnight since the majority of egg-laying occurs in the evening hours [48]. The following morning, females were removed from the arenas by CO_2_ anaesthetization. The oviposition caps were removed and the number of eggs on each cap was counted under a stereoscope at 1.6 × magnification.

### (f) Preseeding

**Fig 1:**
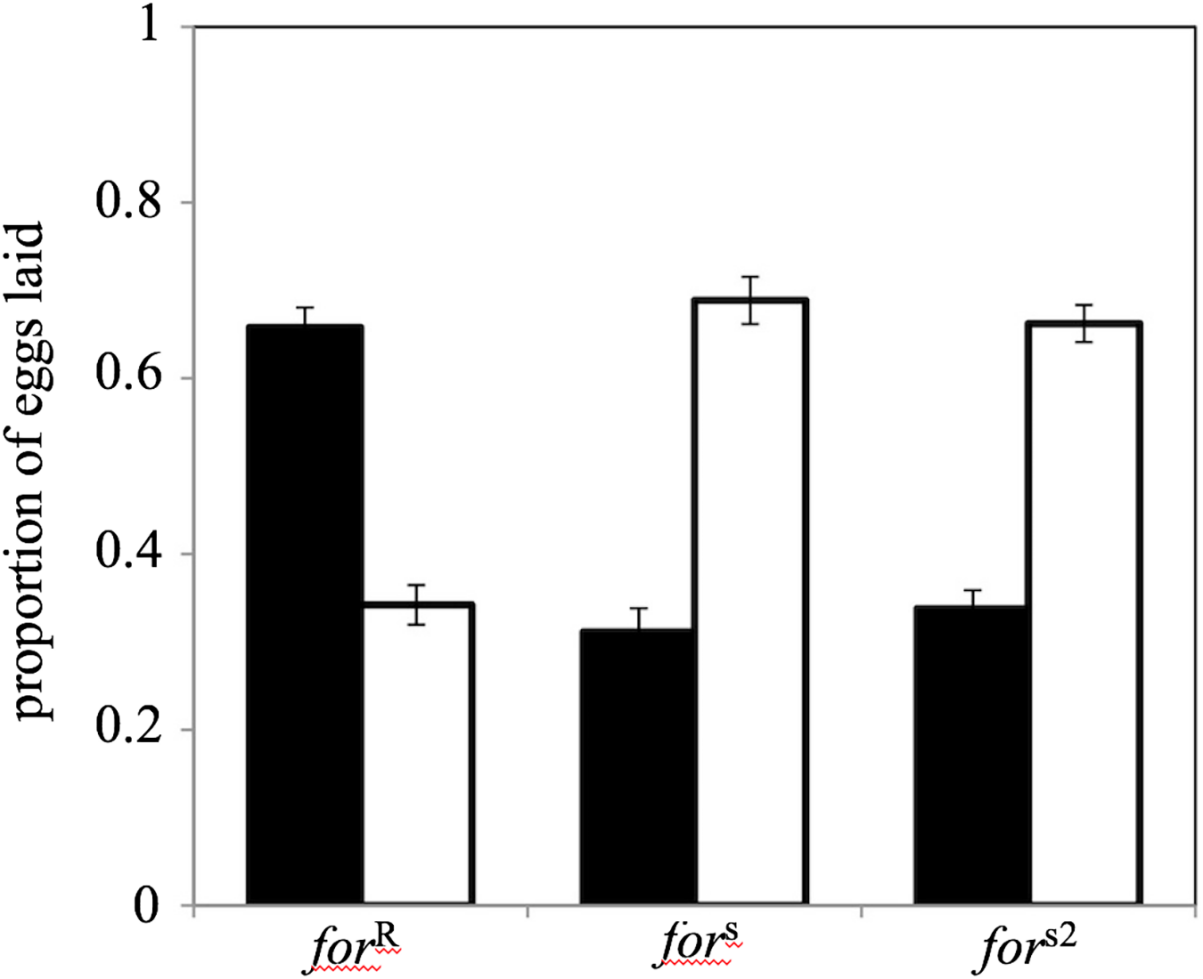
Oviposition preferences are mediated by *for*. *for*^R^ flies preferentially lay on low-nutrient substrates whereas both *for*^s^ and *for*^s2^ flies preferentially lay on high-nutrient substrates. Dark bars represent low-nutrient patches and open bars represent high-nutrient patches. Values represent the mean ± s.e.m. *n* = 20 replicate trials per genotype.

To assess whether the presence of another genotype’s eggs affected the subsequent egg-laying preferences of rovers and sitters, we ‘preseeded’ oviposition food patches with either rover or sitter eggs in either a ‘rover’ or ‘sitter’ pattern. This scenario has potential relevance to the natural world since flies are known to prefer oviposition sites that have been previously occupied [9, 10, 11]. The preseeded eggs were marked with GFP (+; *for*^s^; *Ubi*-GFP and +; *for*^R^; *Ubi-*GFP) to facilitate ease of differentiation between focal (unmarked) and preseeded (marked) eggs. Eggs were obtained by allowing GFP females to oviposit on a grape juice-agar medium (grape plates) overnight at 25 ± 1°C, 65 ± 5% relative humidity (sensu [49]). The next morning, adults were removed and egg-laden grape plates were covered and placed at 4°C. The following day, between 13:00 and 14:30 h, 40 eggs were transferred to experimental food caps using a probe. Four treatments were tested using all four possible combinations of preseeding genotype (rover/sitter) and egg-laying pattern (rover/sitter). Egg-laying pattern was determined using the rover/sitter egg-laying proportions laid on high- and low-nutrient food seen in Fig 1. A density of 40 preseeded eggs was maintained across all combinations. Given that rovers lay approximately 65% of their eggs on low-nutrient food and sitters lay approximately 65% of their eggs on high-nutrient food (Fig 1), eggs were distributed in a 26:14 ratio with L:H representing rover-like OSS and H:L representing sitter-like OSS. An equal number of eggs were distributed haphazardly on each of the two patches per food type. Following this preseeding step the oviposition assay was carried out as described above.

### (g) Effect of nutrient abundance on the survival of rovers and sitters

32 first instar larvae of a given genotype were competed in vials (50 mL, VWR) containing 6 mL of either low- or high-nutrient food. Specific methods for harvesting larvae and conducting the competition experiments can be found in [46].

### (h) Analyses

Normality was assessed using Wilk-Shapiro statistics and since there were no significant deviations from normality we proceeded with parametric analyses. We used arcsine square root transformations for all statistical analyses involving proportions [50]. We display the untransformed proportions in the figures for ease of interpretation. In the experiment comparing the proportion of eggs laid on low-nutrient substrate of rovers, sitters and the sitter mutant (Fig 1), we used a One-Way ANOVA to test the main effects of genotype on egg-laying preferences. We conducted a similar One-Way ANOVA on preferences for low-nutrient substrate when assessing proportion of eggs laid in the transgenic manipulation experiment. In the preseeding egg experiment, we conducted a Three-Way ANOVA to test for the main effects of ovipositing (i.e. focal) genotype (rover/sitter/sitter mutant), preseeded egg type (rover/sitter), and preseeded pattern (rover/sitter) and all possible interactions. All statistical tests were performed using JMP 8.0 (SAS Institute).

## Results

### (a) Role of the foraging gene in rover/sitter OSS

We were unable to detect any significant effect of ‘patch’ for the three genotypes (ANOVA, *F*_1,475_ = 0.04, *p* = 0.85) which indicated that the flies were not clumping their eggs on specific food caps. Consequently, we pooled both patches per food type together for this and all future analyses. We found that rovers lay a significantly greater proportion of their eggs (66 ± 2%, mean ± s.e.m., *n* = 20 trials) on low-nutrient food compared to sitters (31 ± 3%, *n* = 20 trials) and the sitter mutant (34 ± 2%, *n* = 20 trials) (Fig 1; *F*_2,117_ = 56.30, *p* < 0.0001, Tukey *post hoc*). We also found that rovers lay significantly more eggs on average per trial (66.25 ± 5.03, *n* = 20 trials) than sitters (23.60 ± 3.00, *n* = 20 trials) and sitter mutants (32.20 ± 2.54, *n* = 20 trials) (*F*_2,117_ = 37.43, *p* < 0.0001, Tukey *post hoc*).

### (b) Neuronal increase of for-PKG leads to rover-like oviposition preference in sitters

**Fig 2:**
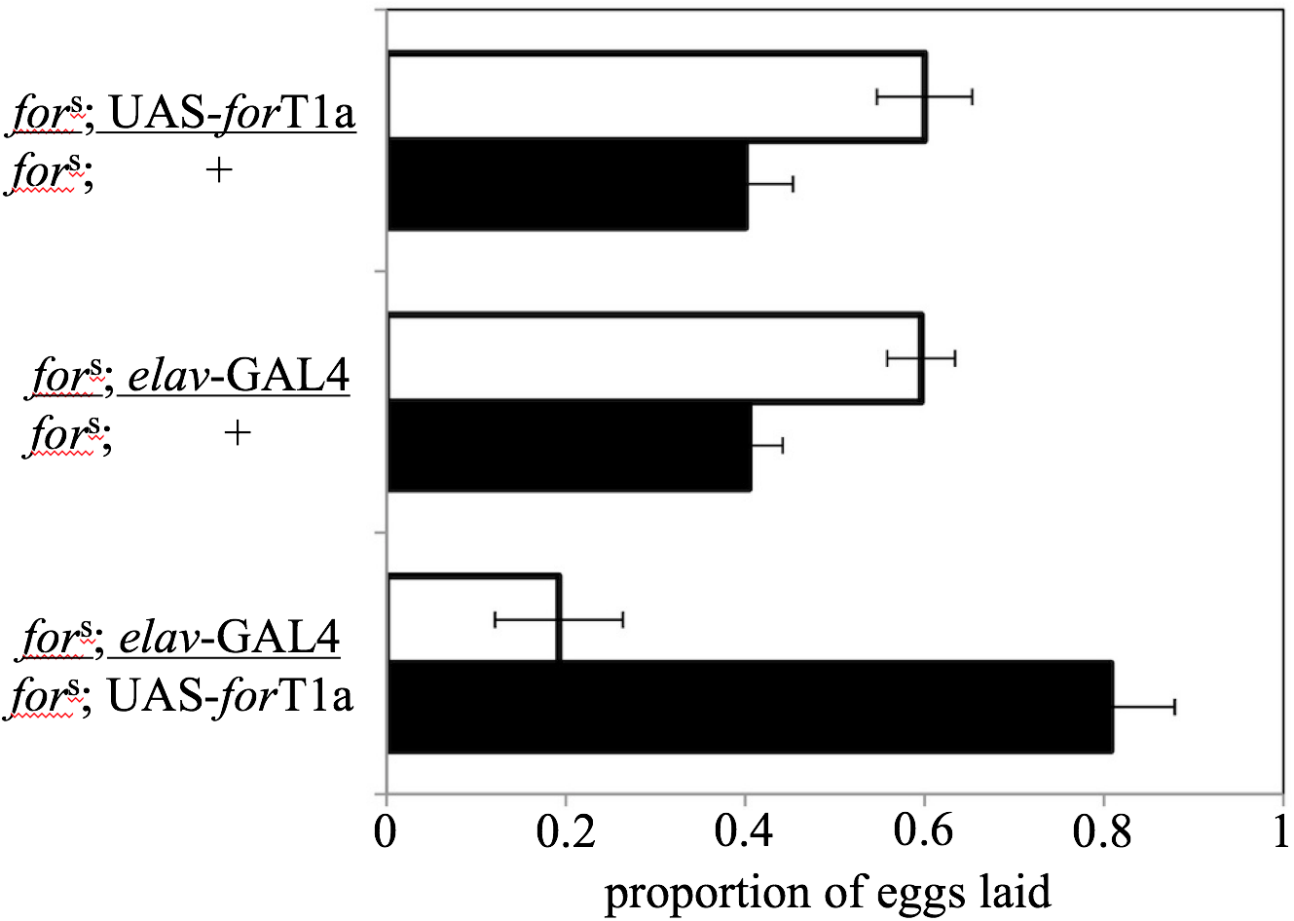
Overexpression of *for* in neurons induces *for*^R^-like oviposition preferences in *for*^s^ flies. The propensity of sitters to ovposit on low nutrient substrates is increased by transgenic overexpression of *for* in their nervous system using the panneuronal *elav*-GAL4 driver (*for*^s^; *elav*-GAL4/UAS-*for*T1a). Control crosses containing only GAL4 (*for*^s^; *elav*-GAL4/+) or UAS (*for*^s^; UAS-*for*T1a/+) had sitter-like oviposition preferences. Dark bars represent low-nutrient patches and open bars represent high-nutrient patches. Values represent the mean ± s.e.m. *n* = 20 replicate trials per genotype.

Sitter flies that overexpressed *for* in their brain and nervous system via the panneuronal *elav* driver (*w*^*1*^; *for*^s^; *elav-*GAL4/UAS- *for*T1a) showed rover-like oviposition preferences. They laid a significantly greater proportion of their eggs on the low-nutrient food (81 ± 7%, *n* = 20 trials), than either of the two experimental controls: w^1^; *for*^s^; *elav-*GAL4/+ (40 ± 3%, *n* = 20 trials) or w^1^; *for*^s^; UAS- *for*T1a/+ (40 ± 5%, *n* = 20 trials) (Fig 2; *F*_2,60_ = 16.73, *p* < 0.0001, Tukey *post hoc*).

### (c) Preseeding results

**Fig 3:**
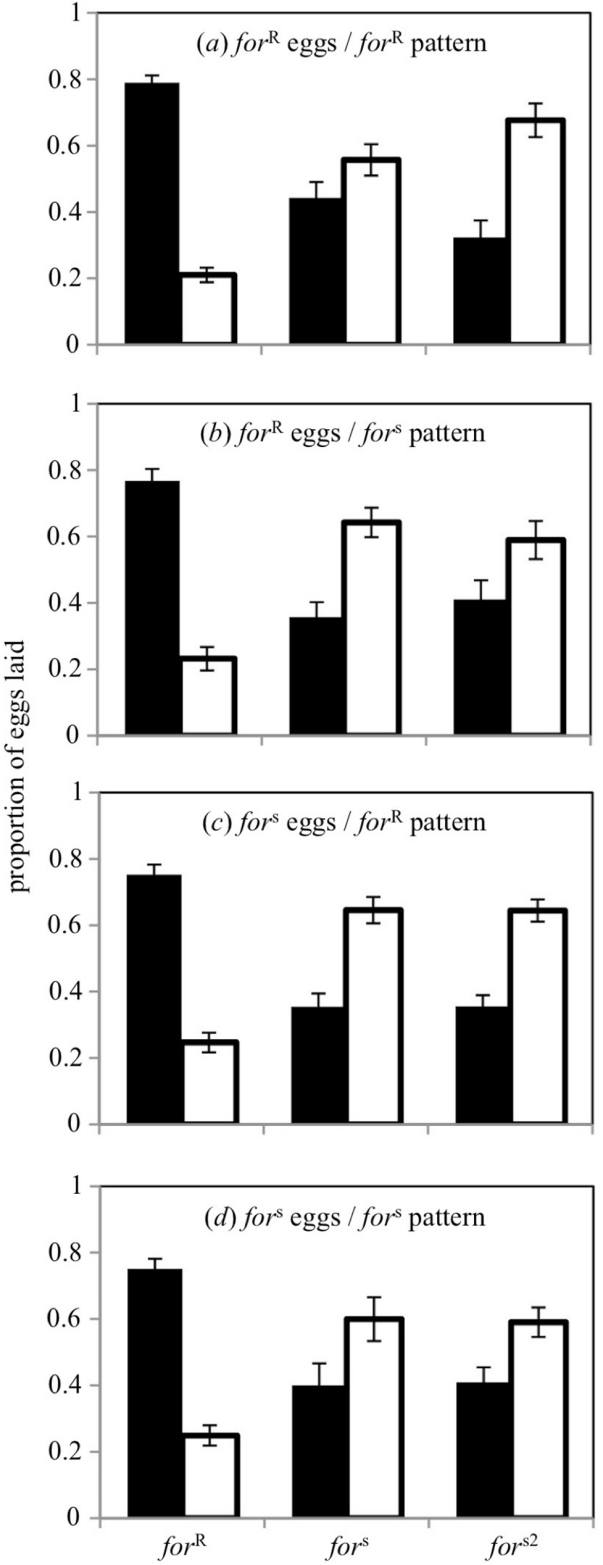
Genotype-specific oviposition preferences are independent of conspecific eggs. *for*^R^ flies consistently lay more eggs on low-nutrient patches regardless of the presence of either *for*^R^ or *for*^s^ eggs preseeded on the patches. Similarly, *for*^s^ and *for*^s2^ preferences for high-nutrient patches are also maintained. Dark bars represent low-nutrient patches and open bars represent high-nutrient patches. Values represent the mean ± s.e.m. *n* = 20 replicate trials per genotype, per condition.

We initially conducted a Three-Way ANOVA (*F*_11, 228_ = 17.00, *p* < 0.0001) that considered the main factors of focal genotype, preseeded genotype, preseeded pattern, and all possible interactions and we failed to detect a significant effect for all variables and all interactions (0.23 ≤ *p* ≤ 0.87) except for the main effect of ovipositing genotype (*p* < 0.0001). Consequently, we reduced the model to a One-Way ANOVA looking only at the effect of ovipositing genotype and found that rovers, across all treatments, laid a significantly greater proportion of their eggs on low-nutrient food than sitters and the sitter mutant (Fig 3; *F*_2,237_ = 91.71, *p* < 0.0001, Tukey *post hoc*). This supports the previous findings reported above (see Fig 1). Given the differences in fecundity between rovers and sitters noted above, we conducted a Three-Way ANOVA using the total number of eggs as the independent variable (*F*_11,228_ = 18.70, *p* < 0.0001) and found significant main effects of both preseeded egg type (*p* = 0.04) and ovipositing genotype (*p* < 0.0001). All interactions were not statistically significant although the three-way interaction of ovipositing genotype × preseeded egg type × preseeded pattern was marginally non-significant (*p* = 0.06). Following this we removed all interactions along with the main effect of pattern and reduced the model to a Two-Way ANOVA looking at the main effects of ovipositing genotype and preseeded egg type. We again found that rovers laid significantly more eggs (80.46 ± 5.24 eggs, *n* = 80 trials) than sitters (23.31 ± 1.7 eggs, *n* = 80 trials) and sitter mutants (30.01 ± 1.77 eggs, *n* = 80 trials) (Two-Way ANOVA, *F*_2,236_ = 88.55, *p* < 0.0001). We also found that the genotypes laid marginally significantly more eggs when in the presence of sitter eggs (rover: 89.68 ± 8.75 eggs, *n* = 40, sitter: 22.83 ± 2.68 eggs, *n* = 40 and sitter mutant: 32.96 ± 2.81 eggs, *n* = 40, respectively) than when in the presence of rover eggs (rover: 71.25 ± 5.50 eggs, *n* = 40, sitter: 23.80 ± 2.19 eggs, *n* = 40 and sitter mutant: 27.05 ± 2.07 eggs, *n* = 40, respectively) (Two-Way ANOVA, *F*_1,236_ = 4.13, *p* = 0.04). However, this marginally significant effect of preseeding egg type does not survive a Bonferroni correction that accounts for multiple comparisons where the critical alpha value is revised to 0.025 (i.e. 0.05/2).

### (d) Effect of nutrient abundance on the survival of rovers and sitters

**Fig 4:**
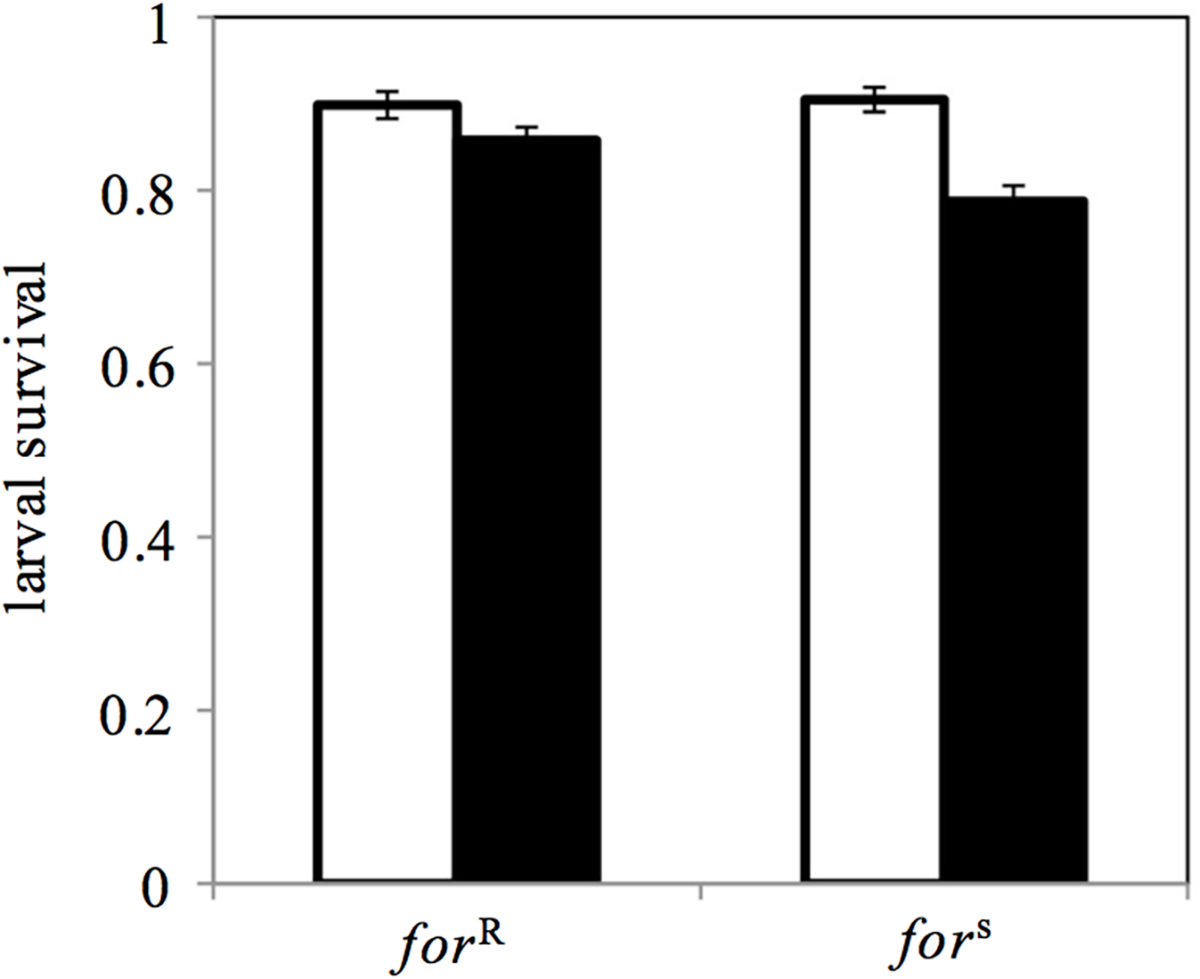
Effect of nutrient availability on rover (*for*^R^) and sitter (*for*^s^) fitness. Rovers were more fit (expressed as the proportion surviving to pupation) than sitters when reared on low-nutrient food. Fitness was higher and the genotypes were indistinguishable when reared on the high-nutrient food. Dark bars represent low-nutrient conditions and open bars represent high-nutrient conditions. Values represent the mean ± s.e.m., *n* = 20 replicate trials per genotype, per food type.

Fitness, the proportion surviving to pupation, was calculated for each vial (*n* = 20 per genotype per food condition). Proportions were arcsine squareroot transformed prior to analyses [50]. We used a Two-Way ANOVA to assess the role of genotype (rover, sitter) and nutrient abundance (low- and high-nutrient) on fitness and found a significant effect of nutrient abundance (*p* < 0.0001) and the nutrient × genotype interaction (*p* = 0.04) but no significant effect of genotype (*p* = 0.11)(*F*_3,76_ = 10.33, *p* < 0.0001, Fig 4). Given the interaction, we analysed the two food types separately using Students t-tests. We found that rovers had higher fitness (0.86 ± 0.02) than sitters (0.79 ± 0.02) on low-nutrient food (t = 2.98, df = 37.95, *p* = 0.005). However, the survival of rovers and sitters was statistically indistinguishable when reared on high-nutrient food (rover: 0.90 ± 0.02, sitter: 0.90 ± 0.02) (t = 0.30, df = 38, p = 0.76).

## Discussion

Recent evidence from Miller *et al*. [14] shows there is natural variation in the egg-laying preferences of fruit flies. The results from our study suggest that naturally occurring genetic variation in *foraging* is a strong contributor to population-level variation in OSS. Rover flies, characterized by more exploratory foraging behaviour, lay a significantly greater proportion of their eggs on low-nutrient substrates whereas sitters and the sitter mutant (*for*^*s2*^) show significantly greater preference for high-nutrient substrates. In addition to the *for*^s2^ mutation, we further confirm a role for the *for* gene from our observations when *for* expression is increased transgenically in sitters to rover-like *for* transcript and PKG protein levels [24, 31], and rover-like egg-laying preferences. Results from the preseeding experiment suggests that rover/sitter egg-laying decisions are not influenced by social cues (previously laid eggs).

The PKG molecule encoded by *for* is a major signaling molecule with many downstream effects (reviewed in [51]). PKG functions by phosphorylating serine and threonine residues on a diverse array of proteins [52] and is responsible for major cellular processes such as signal transduction, muscle relaxation, nociception, and platelet function [53, 54]. The effect of *for* on behaviour was first described on larval foraging [20, 23] and in recent years this has also been extended to several other phenotypes (see Introduction). This vast assortment of pleiotropic effects associated with *for* is likely facilitated by the diverse role of PKG at the molecular and cellular level [54].

At the physiological level, the specific substrate preferences of rovers and sitters may be rooted in taste. Previous work has shown that *for*-PKG modulates sensitivity to sugar [30, 31] wherein sitters are less responsive to sucrose than rovers. The low- and high-nutrient food types used on our study differed in both yeast and sugar levels. Therefore, it is possible that, in addition to differences in sugar sensitivity, rovers and sitters may differ in other tastants associated with yeast such as bitter and umami [55, 56]. Therefore, necessary future work includes experiments that explicitly address the notion of taste underlying rover/sitter OSS, and that dissect the specific preference cues.

According to our results, rover flies lay a greater proportion of their eggs in a sub-optimal environment compared to sitters. In the proceeding paragraphs we suggest that the: i) better nutrient absorption, ii) higher fecundity, and iii) greater inter patch movement and dispersal of rovers compared to sitters may explain these egg-laying differences.

Firstly, the low-nutrient substrate used in our experiment is known to have a significant impact on both rover and sitter fitness as evidenced by an increased development time and reduced survivability relative to the high nutrient substrate ([32, 46], Fig 4). However, work by Kaun *et al*. [32] has also shown that rovers are better able to absorb carbon from glucose than sitters despite the fact that rovers consume less food than sitters during a given period of time. This difference in absorption and metabolism in rovers and sitters is possibly evidenced by the fact that despite an overall fitness decrease due to the low-nutrient food (Fig 4; ANOVA, *F*_3,76_ = 10.33, *p* < 0.0001) there was a nutrient 0078 genotype interaction whereby rovers survived significantly better than sitters on the low-nutrient food but both survive equally well on high-nutrient food (interaction, *p* = 0.04). Thus, it is possible that this ability to better manipulate and utilize a typically ‘suboptimal’ source allows rover females to exploit a nutrient poor egg-laying substrate at minimal cost to offspring fitness.

Secondly, in our experiments we found that rovers laid approximately 2.8 times as many eggs as sitters. Therefore, the lower survivorship associated with the low-nutrient substrate may be compensated in rovers by their increased fecundity.

Finally, although our results show differences in where eggs are laid, the developmental life history of those larvae in nature is unknown. Larvae developing in nature experience heterogeneity in food patches, heterogeneity of food sources (fruits), predators, and parasitoids. From laboratory studies we know that rover larvae regularly leave food patches and explore new food patches whereas tend to stay on a single food patch [57]. It is therefore possible that the egg-laying decisions we observed do not directly correlate with the ultimate location of development of the emergent larvae. Given the tendency of sitters to remain on a food patch, it may be important for sitters to hatch on a substrate that can support their full development. Laying on a higher nutrient substrate, which may represent an optimally fermenting ripe fruit in nature, will satisfy this goal. However, this may also come with consequences including a high probability of competition with other larvae, competition with other organisms, and greater exposure to predators and parasitoids. Rover egg-laying decisions may reflect a means to reduce these consequences since the low-quality food in our experiments might be analogous to a newly fermenting, less ripe fruit (lower yeast and sugar availability) having few larvae and predators. Following hatching, the greater foraging and exploratory tendencies of rovers could allow them to optimally adjust their feeding location.

Our study exposes the necessity for more field-based studies looking at the movement of rover and sitter larvae in natural environments. Little is known about the life history of rovers and sitters in nature despite a wealth of laboratory-based studies. Understanding the natural range in ‘quality’ of food patches (nutrient availability), the distribution of food patches and food sources, and the natural ranges in larval density and competition would greatly help to put the findings of our study into a more natural context.

To fully understand the fitness consequences of the observed rover and sitter egg-laying preferences, future work should look at larval survivorship when reared in proportions similar to egg-laying preferences. This will allow for a greater understanding of whether rover/sitter differences in oviposition preferences have effects on offspring fitness and the role of frequency-dependent selection in maintaining the rover/sitter polymorphism [46].

With multiple lines of evidence, our study shows that naturally varying expression levels of the *for* gene influences egg-laying decisions. Rover flies, with naturally higher expression of *for*, lay a significantly greater proportion of eggs on low-nutrient food when compared with sitters and the sitter mutant, each with naturally lower *for* expression. This was verified by assaying transgenic crosses for oviposition preferences using the panneuronal driver *elav*-GAL4 to overexpress *for*-mRNA in sitter flies to rover-like levels. Indeed, when *for*-mRNA is increased in sitters, their oviposition preferences changes to that of a rover. Our study further dissects oviposition behaviour in *D*. *melanogaster* by both looking at the mechanisms underlying observed egg-laying preferences as well as inferring the ecological reasons as to why such differences exist in nature.

## Supporting information

S1 File Data used in this paper. This file contains the data from this paper.
(XLSX)
